# Combined effects and weight analysis of factors affecting initial maxillary central incisor retraction: a three-dimensional finite element study

**DOI:** 10.3389/fbioe.2026.1895763

**Published:** 2026-09-07

**Authors:** Feng Feng, Bin Xing, Yucheng Wang, Yuheng Liu, Yicong Liu, Wen Liu

**Affiliations:** 1 Department of Stomatology, Qingdao Hospital, University of Health and Rehabilitation Sciences (Qingdao Municipal Hospital), Qingdao, Shandong, China; 2 Department of Stomatology, Qingdao Women and Children’s Hospital, Qingdao University, Qingdao, Shandong, China; 3 Department of Orthodontics, Qingdao University Affiliated Hospital, Qingdao, Shandong, China; 4 The State Key Laboratory of Computer Aided Design and Computer Graphics (CAD&CG), College of Computer Science and Technique, Zhejiang University, Hangzhou, China; 5 Department of Stomatology, Qingdao Huanghai University Stomatological Hospital, Qingdao, Shandong, China; 6 Department of Stomatology, Qilu Hospital (Qingdao), Cheeloo College of Medicine, Shandong University, Qingdao, Shandong, China

**Keywords:** alveolar bone loss, bone density, finite element analysis, regression analysis, tooth movement techniques

## Abstract

**Introduction:**

Investigating the stress distribution generated by orthodontic forces within the periodontal ligament (PDL) is critical for quantifying mechanical stimuli, correlating them with subsequent histological responses, and ultimately identifying evidence-based optimal force levels. This study aimed to evaluate the combined biomechanical effects of alveolar bone height loss, tooth movement type, and alveolar bone density on the initial retraction of the maxillary central incisor, and to quantify the weighting of each factor on PDL stress.

**Methods:**

A three-dimensional (3D) finite element model of the maxilla, dentition, PDL, and orthodontic appliances was constructed based on cone-beam computed tomography (CBCT) data. Eighty experimental conditions were established by combining five levels of alveolar bone height loss (0–4 mm), four tooth movement types, and four alveolar bone density levels (simulated via cortical bone Young’s modulus: 12,500–27,500 MPa). A 1 N bilateral retraction force was applied. The initial displacements of the crown and root and the maximum PDL von Mises stress were calculated. Multiple linear regression was used to quantify the factor contributions.

**Results:**

Alveolar bone height loss significantly increased both tooth displacement and PDL stress. Uncontrolled and controlled tipping resulted in greater sagittal retraction and root intrusion, whereas bodily and controlled root movements were associated with more distolingual rotation. However, bone density affected displacement but had no significant effect on PDL stress. The regression model (R^2^ = 0.80) revealed that tooth movement types exerted the strongest influence on PDL stress, followed by alveolar bone height loss (β = 0.535, *p* < 0.05). All conditions, except for 4 mm bone loss combined with uncontrolled tipping, exhibited PDL stresses below the 0.026 MPa safety threshold.

**Conclusion:**

These findings provided novel quantitative biomechanical evidence regarding the combined effects of these variables and could guide individualized force control strategies and the development of AI-assisted orthodontic decision-making systems.

## 1 Introduction

Orthodontic forces are transmitted to teeth and their surrounding periodontal tissues, including the periodontal ligament (PDL) and alveolar bone. The resulting stress distribution within the PDL is recognized as a key initiating factor in tissue remodeling, as it determines the type and magnitude of cellular responses and thereby dictates the rate of tooth movement. This remodeling process produces microscopic tissue displacements, the accumulation of which over time manifests as clinically observable tooth movement (Choi et al., 2016; Rudolph et al., 2001). Appropriate forces can facilitate physiological alveolar bone remodeling and ensure steady tooth movement at an optimal rate, thereby preventing movement stagnation and prolonged treatment duration that would otherwise result from inappropriate force application (Melsen, 2001). Therefore, precise control of orthodontic force is essential for achieving successful orthodontic treatment outcomes. However, the optimal force level is influenced by multiple factors. In addition to the magnitude of the clinically applied force, other factors (e.g., alveolar bone height loss, tooth movement type, and alveolar bone density) also play important roles (Sia et al., 2009).

Previous studies have investigated the contributions of these factors in isolation. For example, Jeon et al. have demonstrated that alveolar bone height loss enhances PDL stress under the same load compared with conditions without bone loss (Jeon et al., 2001). Tanne et al. have investigated the relationship between moment-to-force (M/F) ratios and the center of rotation (CRot) during tooth movement (Tanne et al., 1988). Peng et al. have used near-infrared vibration to modulate alveolar bone density and accelerate tooth movement (Peng et al., 2026). Notably, many studies have shown that light and heavy orthodontic forces produce comparable effects during the initial phase of tooth movement (Yee et al., 2009). Despite these findings, current studies on the combined effects of these factors remain limited. In clinical practice, the control of orthodontic force often depends on the clinician’s experience and subsequent observation of tooth movement—an approach that is essentially a continuous trial-and-error process that significantly affects orthodontic efficiency and treatment outcomes.

With the rapid development of artificial intelligence (AI), machine learning (ML)—a core branch of AI—offers hope for achieving more precise and efficient medical care. ML algorithms can objectively analyze large datasets, identify key influencing factors, and build predictive models by quantifying the relative weight of each factor, thereby supporting clinical decision-making and individualized treatment planning (Likitmongkolsakul et al., 2018; Trakulmututa and Win, 2026). Investigating the stress distribution of orthodontic forces within the PDL is critical for quantifying mechanical stimuli, linking them to subsequent histological responses, and ultimately defining evidence-based optimal force levels (Paddenberg-Schubert et al., 2025). By analyzing the effects of various factors on PDL stress distribution and quantifying their respective contribution weights, we can incorporate these weights into orthodontic diagnosis, treatment planning, and clinical management, and also develop ML-based decision-support systems via the development of robust predictive models.

Positioned at the most anterior region of the dental arch, the maxillary central incisor is highly susceptible to orthodontic forces and is prone to adverse effects such as root resorption, bone dehiscence, and fenestration (Hong et al., 2024; Li et al., 2024). As a high-precision and non-destructive technique for structural stress analysis, finite element modeling is widely used to simulate biomechanical responses in orthodontics (Ma et al., 2025). Because the initial tooth movement caused by orthodontic force is extremely small during the early stage of loading, the PDL can be approximately regarded as a linear-elastic material (Geramy, 2002). In the present study, finite element modeling was used to systematically simulate the biomechanical environment during maxillary anterior retraction following first premolar extraction and to examine the initial PDL stress response. The objective of this study was to assess the combined effects of the three factors on the initial retraction of the maxillary central incisor, and to quantitatively evaluate the contribution of each factor to the overall biomechanical response of the PDL.

## 2 Materials and methods

### 2.1 Three-dimensional (3D) finite element modeling

A healthy 20-year-old adult with normal occlusion was selected according to the following criteria: (1) complete dentition without malocclusion; (2) healthy periodontal status without clinically significant alveolar bone resorption; (3) absence of carious lesions or clinically significant tooth attrition; (4) no history of orthodontic or orthognathic surgery; and (5) absence of systemic metabolic diseases. The study protocol was approved by the Ethics Committee of Qingdao Municipal Hospital (approval no. 2025-KY-091 [for speedy trial]) and was conducted in accordance with the Declaration of Helsinki and its later amendments, as well as with comparable ethical standards. Written informed consent was obtained from the volunteer.

The volunteer was scanned using a cone-beam computed tomography (CBCT) scanner (KaVo, Germany) at a slice thickness of 0.15 mm, generating a total of 576 images. During the procedure, the volunteer was instructed to remain stationary and to refrain from swallowing or taking deep breaths. The acquired imaging data, covering both jaws and the complete dentition, were stored in DICOM format and then imported into Mimics software (Materialise, Belgium). Based on differences in Hounsfield unit thresholds between hard and soft tissues in the head region, appropriate thresholds were set to separate bone from soft tissue, thereby generating initial 3D models of the maxillary dentition and the maxilla. The resulting STL file was then imported into Geomagic Studio (Hexagon, Sweden) for surface optimization, after which the entire maxillary section was offset inward by 1 mm. Cortical and cancellous bone were subsequently generated via Boolean operations in SolidWorks (Dassault, France). Given that the PDL appeared as a heterogeneous structure in the DICOM data and could not be completely separated using the software, the corresponding teeth were duplicated in Geomagic Studio following previous methods and then offset outward by 0.25 mm. Excess portions were then removed in SolidWorks with the “Split” command, and Boolean operations were performed to generate the 3D PDL model. In SolidWorks, a maxillary archwire with a cross-section of 0.48 mm × 0.64 mm (0.019 in × 0.025 in) was constructed. Additionally, simplified brackets with a slot size of 0.56 mm × 0.71 mm (0.022 in × 0.028 in) and two simplified hooks with a height of 2 mm were generated. The previously established solid models of the maxillary cortical bone, cancellous bone, and dentition were then imported. Bracket positions were adjusted to align with the labial surfaces of the crowns, and the archwire was inserted into the bracket slots. Two traction hooks were placed bilaterally at the midpoint of the archwire between the lateral incisor and the canine. In this model, the archwire was assumed to be passively inserted into the bracket slots after complete leveling. The final assembly was then exported in IGES format.

### 2.2 Model groups

#### 2.2.1 Alveolar bone height loss

**FIGURE 1:**
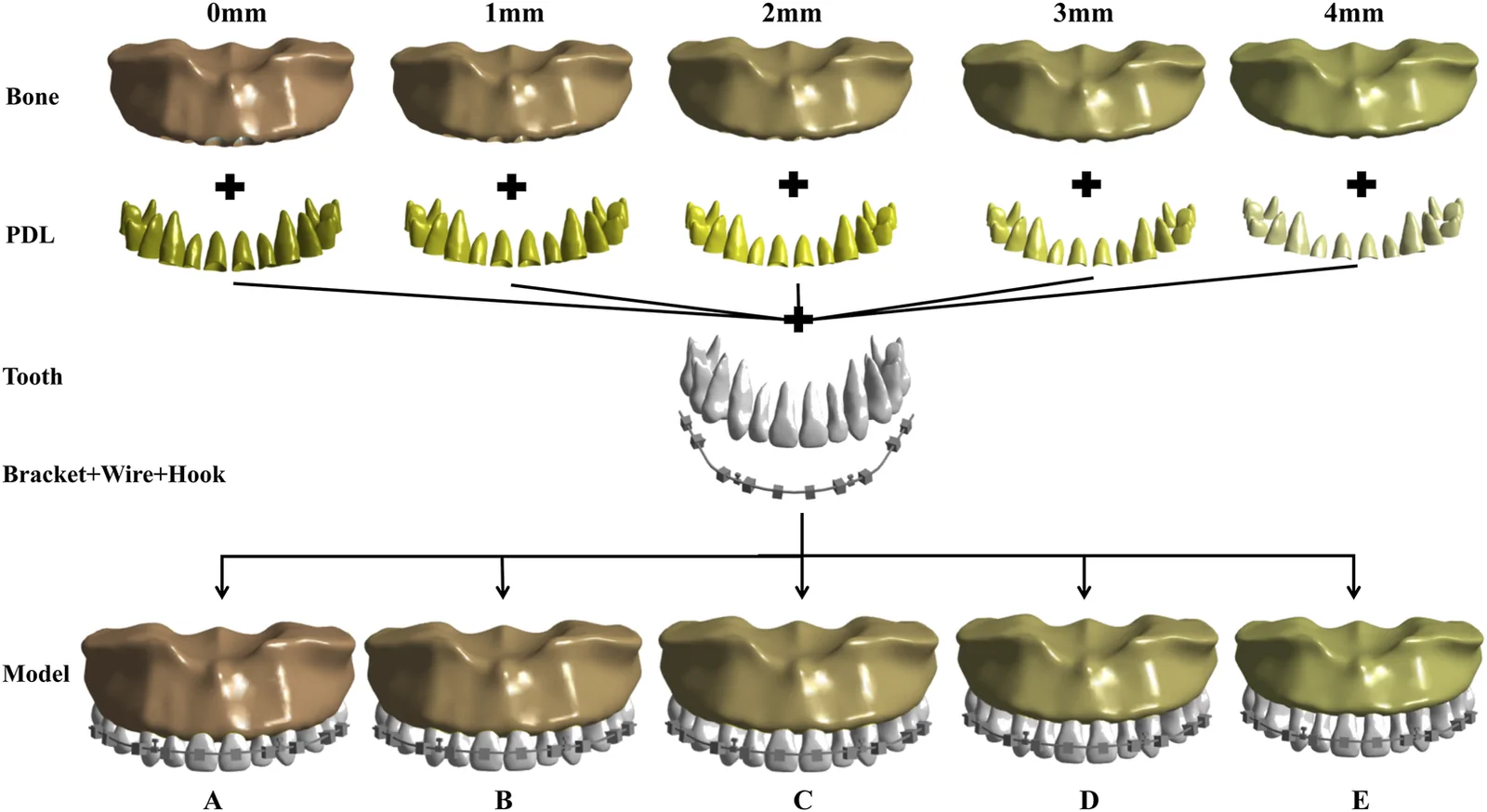
Modeling process of 3D finite element models for anterior tooth retraction after first premolar extraction. Five models were individually assembled: normal (Model A), 1 mm alveolar height bone loss (Model B), 2 mm alveolar height bone loss (Model C), 3 mm alveolar height bone loss (Model D), and 4 mm alveolar height bone loss (Model E).

It has been demonstrated that the height of the alveolar bone is considered one of the critical determinants of the limits for tooth movement, and its loss further restricts the boundary of tooth movement (Ahn et al., 2013). Alveolar bone height loss results from factors such as age-related changes, skeletal pattern, systemic diseases, and faulty restorations, and is also regarded as the most important clinical manifestation of periodontitis (Jäger et al., 2017; Luo et al., 2024; Schubert et al., 2020; Tang et al., 2024). Horizontal alveolar bone loss has been shown to be more prevalent than vertical bone loss and has a more pronounced effect on stress outcomes (Zasčiurinskienė et al., 2023). Therefore, in our study, five models with different simulated alveolar bone heights were established. Using the offset-merge method in Geomagic Studio, the bone surface was uniformly offset inward relative to the model with normal bone height. This resulted in alveolar bone height reductions of 1, 2, 3, and 4 mm. The resulting files were then imported into SolidWorks. The PDL corresponding to the teeth in each model was generated using Boolean operations. The dentition, brackets, archwire, and traction hooks remained unchanged across all models. Five models—normal (Model A), 1 mm alveolar bone height loss (Model B), 2 mm alveolar bone height loss (Model C), 3 mm alveolar bone height loss (Model D), and 4 mm alveolar bone height loss (Model E)—were assembled individually and then imported into ANSYS Workbench (Ansys, United States) for static structural analysis (Figure 1).

#### 2.2.2 Tooth movement types

**FIGURE 2:**
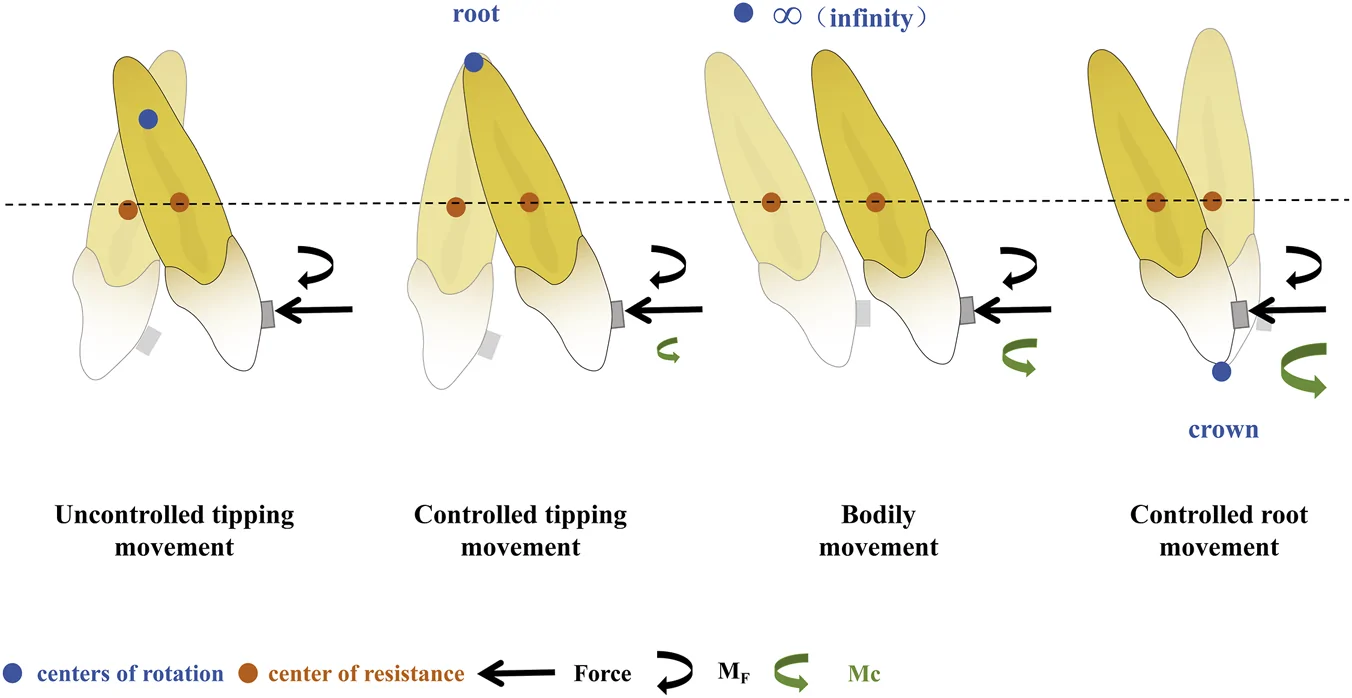
As the applied moment increases, the maxillary central incisor shows four movement types sequentially (from left to right): uncontrolled tipping, controlled tipping, bodily movement, and controlled root movement. M_F_: Moment induced by forces; M_C_: Moment applied in a progressively increasing manner.

To achieve different types of tooth movement, the M/F ratio must be appropriately controlled to position CRot of the tooth at the desired point (Figure 2) (Tang et al., 2025; Tanne et al., 1988). The CRot can serve as an important reference for evaluating the effect of controlled tooth movement, and its location can be estimated from the sagittal displacements of landmarks at the crown and root apex according to Equation 1.

$$D_{\text{CRot}\;}=\frac{\left.\left.L\times \right|D_{\text{root}}\right|}{\left|D_{\text{crown}}\left|+\right.\left|D_{\text{root}\;}\right|\right.}$$ (1)

**FIGURE 3:**
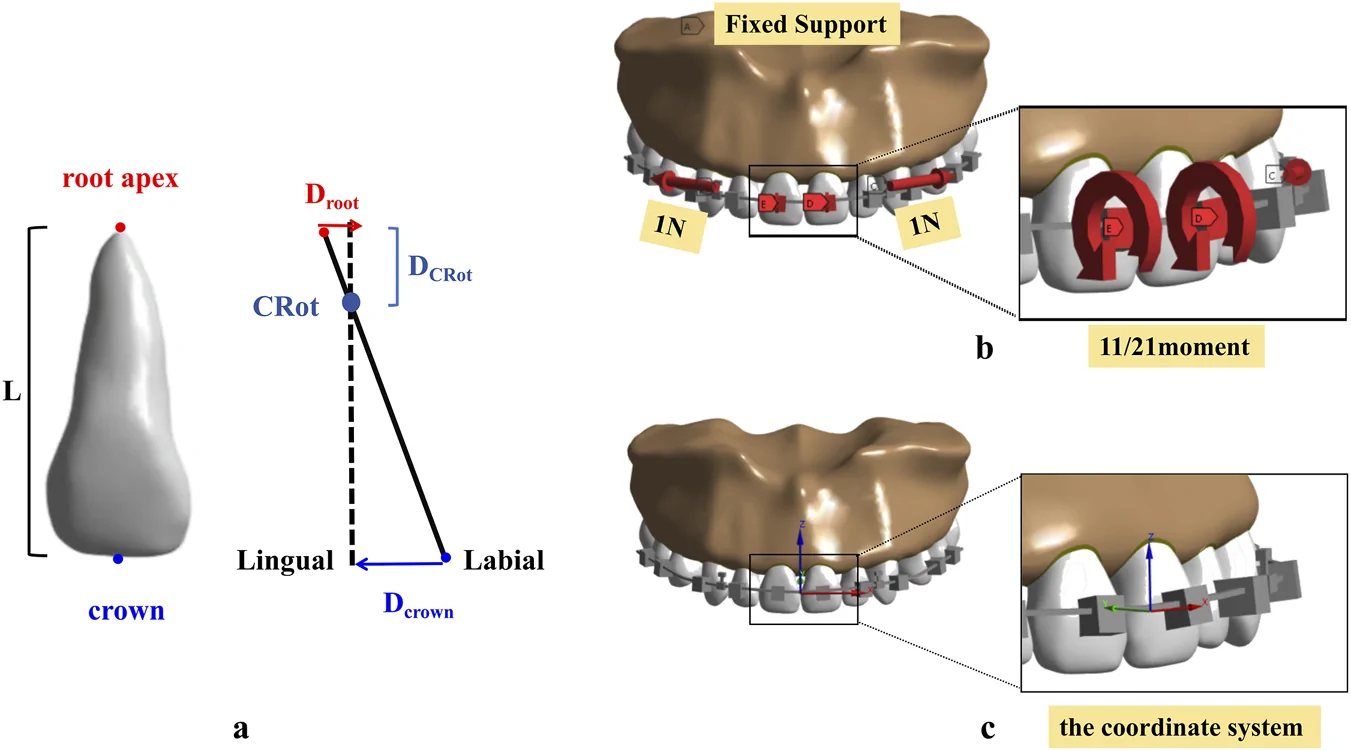
**(a)** Observation values. D_CRot_ is the distance between the CRot and the root apex; L is the tooth length. Crown and root displacement are defined as positive in the labial-to-lingual direction and negative in the lingual-to-labial direction. D_crown_ and D_root_ represent the sagittal displacements of the crown and root landmarks, respectively. The midpoint of the incisal edge of the maxillary central incisor crown is used to represent initial displacement of the crown along the X-, Y-, and Z-axes; the root apex is used to represent initial displacement of the root. **(b)** Schematic diagram of boundary conditions and loading modes in the finite element model. All degrees of freedom are constrained on the superior surface of the maxilla. A force of 1 N per side is applied from the hooks, parallel to the archwire. The loading principle is illustrated to intuitively demonstrate the effect of moment application. **(c)** Establishment of the coordinate system. The X-axis represents the horizontal movement tendency of the tooth (positive to the right), the Y-axis represents the sagittal movement tendency (positive posteriorly), and the Z-axis represents the vertical movement tendency (positive upward).

Where D_CRot_ is the distance between CRot and the root apex, L is the tooth length, and D_crown_ and D_root_ represent the sagittal displacements of the crown and root landmarks, respectively. Displacement in the labial-to-lingual direction is defined as positive, whereas displacement in the opposite direction is defined as negative (Figure 3a) (Tanne et al., 1988; Yoshida et al., 2001).

**FIGURE 4:**
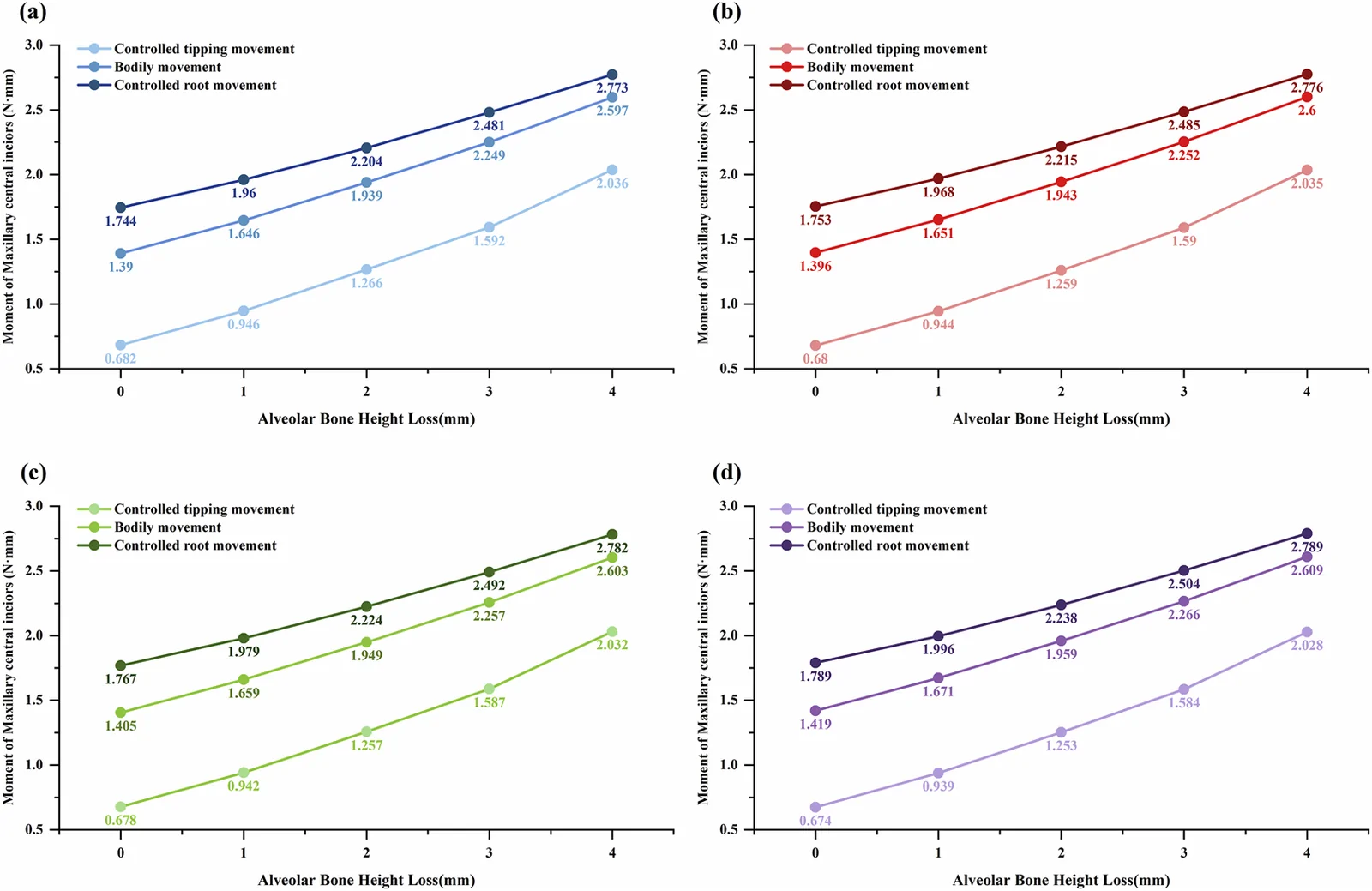
Applied moment on the maxillary central incisors. The labels of 0, 1, 2, 3, and 4 mm in the figure indicate the degree of alveolar bone height loss. **(a–d)** correspond to alveolar bone density levels I–IV respectively.

A retraction force of 1 N per side was applied from the hooks to the first molar, parallel to the archwire, to simulate clinical orthodontic retraction (Figure 3b). This value was chosen to reflect clinically relevant force levels (Proffit et al., 2006) and induced lingual crown tipping of the tooth. Meanwhile, a concentrated counterclockwise moment about the mesiodistal (X) axis was applied to the center point of the maxillary central incisor bracket in ANSYS Workbench to simulate the reaction moment induced by archwire deformation during clinical orthodontics (Wang et al., 2025; Wu et al., 2023) (Figure 3). By incrementally increasing the moment magnitudes shown in Figure 4 and Supplementary Table S1 and estimating CRot based on the sagittal displacements of the crown and root apex landmarks, four types of tooth movement were defined as follows:

1. Uncontrolled tipping: the crown moved lingually whereas the root moved labially.
2. Controlled tipping: the crown moved lingually while the root remained approximately in its original position, with the CRot located at the root apex.
3. Bodily movement: the crown and root moved lingually by nearly equal amounts, and the CRot was situated at infinity.
4. Controlled root movement: both the crown and root moved lingually, with root movement being greater in magnitude than crown movement magnitude, and the CRot was located at the incisal edge.

#### 2.2.3 Alveolar bone density

Bone density is a clinical indicator of bone mass and directly determines the mechanical strength and Young’s modulus of bone tissue. Previous studies have shown that tooth movement is accelerated under lower bone density but slowed under higher bone density due to the deformation resistance of cortical bone (Kuc et al., 2025; Seong et al., 2009). In the present study, variations in alveolar bone density were simulated by adjusting the Young’s modulus of the alveolar cortical bone. Four bone density levels (I–IV), corresponding to high, moderately high, moderately low, and low density, were defined with alveolar cortical bone Young’s modulus values of 27,500, 22,500, 17,500, and 12,500 MPa, respectively. The modulus values were derived from published literature (Supplementary Table S2) (Kuc et al., 2025; Seong et al., 2009).

In total, 80 working conditions were established based on the above classifications.

### 2.3 Material parameter assignment and mesh generation

In consideration of numerical convergence and computational feasibility, all components (maxillary cortical bone, maxillary cancellous bone, PDL, teeth, brackets, traction hooks, and archwire) were assumed to be homogeneous, isotropic, and linearly elastic. This is consistent with established practices in the literature (Choi et al., 2025; Papageorgiou et al., 2017; Xia et al., 2024). Notably, the PDL was modeled as an isotropic, linearly elastic material rather than a hyperelastic material in this study. Specifically, the study simulated the initial loading phase of orthodontic treatment, during which external forces are small and PDL deformation typically falls within the small-strain regime. Under small-strain conditions, the initial tangent stiffness of a hyperelastic material model presents an approximately linear elastic response (Wang et al., 2022). Therefore, the PDL was modeled as a linear elastic material in this study, which is not expected to significantly affect the relative trends across different loading conditions (Wood et al., 2011). Relevant material properties, including Young’s modulus and Poisson’s ratio, were derived from the literature and are summarized in Supplementary Table S2.

To enhance the convergence of the calculations, the 3D models were meshed with 10-node quadratic tetrahedral solid elements in ANSYS Workbench, which are suitable for contact analysis. The model was discretized through meshing, and the numbers of nodes and elements for the five models (Models A–E) are presented in Supplementary Table S3. To ensure mesh independence of the numerical results, a mesh convergence study was performed on the baseline finite element model using four levels of progressive mesh refinement (1.5, 1, 0.8, and 0.5 mm). The maximum von Mises stress was adopted as the convergence metric, and its values across obtained with all mesh densities were compared (Huang et al., 2026). Convergence was considered achieved when the relative difference between two consecutive refinement levels was below 5%. The difference between the 0.8 mm and 0.5 mm meshes was 0.63%, which met the convergence criterion.

### 2.4 Boundary condition setting and establishment of the coordinate system

Fixed boundary conditions were applied to the superior surface of the maxilla, as shown in Figure 3a. Interfaces were defined as bonded contacts, including those between the maxillary cortical bone and cancellous bone, cortical bone and PDL, cancellous bone and PDL, PDL and tooth, tooth and bracket, and traction hooks and archwire. The connections between adjacent teeth were modeled as no-separation contacts; however, small amounts of frictionless sliding are allowed to occur along the contact surfaces (Liu et al., 2022; Zhang et al., 2024). Furthermore, the contact condition between bracket and archwire was also defined as no separation, meaning that the archwire was allowed to slide freely along the bracket slot but could not separate vertically from it. To simulate the effective torque acting on the tooth, any clearance between the bracket and archwire was not considered.

In this study, the midpoint of the archwire segment between the two maxillary central incisors was defined as the coordinate origin. The plane containing the archwire was designated as the X–Y plane, while the plane perpendicular to it was defined as the Y–Z plane. The intersection of these two planes defined the X-, Y-, and Z-axes. The X-axis represented the horizontal movement tendency of the tooth (positive to the right), the Y-axis represented the sagittal movement tendency (positive posteriorly), and the Z-axis represented the vertical movement tendency (positive upward) (Figure 3c).

### 2.5 Observation values and statistical analysis

In ANSYS Workbench, the midpoint of the incisal edge of the maxillary central incisor crown was used to measure initial displacement of the crown along the X-, Y-, and Z-axes; the root apex was used to represent initial displacement of the root (Figure 3a). The initial 3D displacement between the crown and root of the maxillary central incisor and the initial equivalent (von Mises) stress within its PDL were analyzed. Among these, Von Mises stress condenses the 3D stress tensor into a single scalar value to identify stress concentrations, which aligns with our focus on relative stress distribution trends (Huang et al., 2026). Finally, given the bilateral symmetry of the models, data were collected only for the right maxillary central incisor.

Multiple linear regression analysis requires that the dependent variable is continuous. In this study, because the dependent variable (PDL stress) met this criterion, multiple linear regression analysis was conducted to predict von Mises stress in the PDL using alveolar bone height loss, tooth movement types, and alveolar bone density as independent variables. Alveolar bone density and tooth movement types were categorical variables and were therefore converted into dummy variables prior to regression analysis to improve model reliability and predictive accuracy. Notably, multiple linear regression was used as a model-based sensitivity analysis of simulation outputs to quantify the contributions of the independent variables to the dependent variable. Since our 80 observations were deterministic finite element results obtained from a single anatomical model, the regression coefficients and significance levels were interpreted strictly within the simulation framework and did not imply causal relationships. All statistical analyses were performed using Origin Pro 2024b (OriginLab Corporation, United States).

## 3 Results

### 3.1 Crown and root displacement of the maxillary central incisor in three dimensions

**FIGURE 5:**
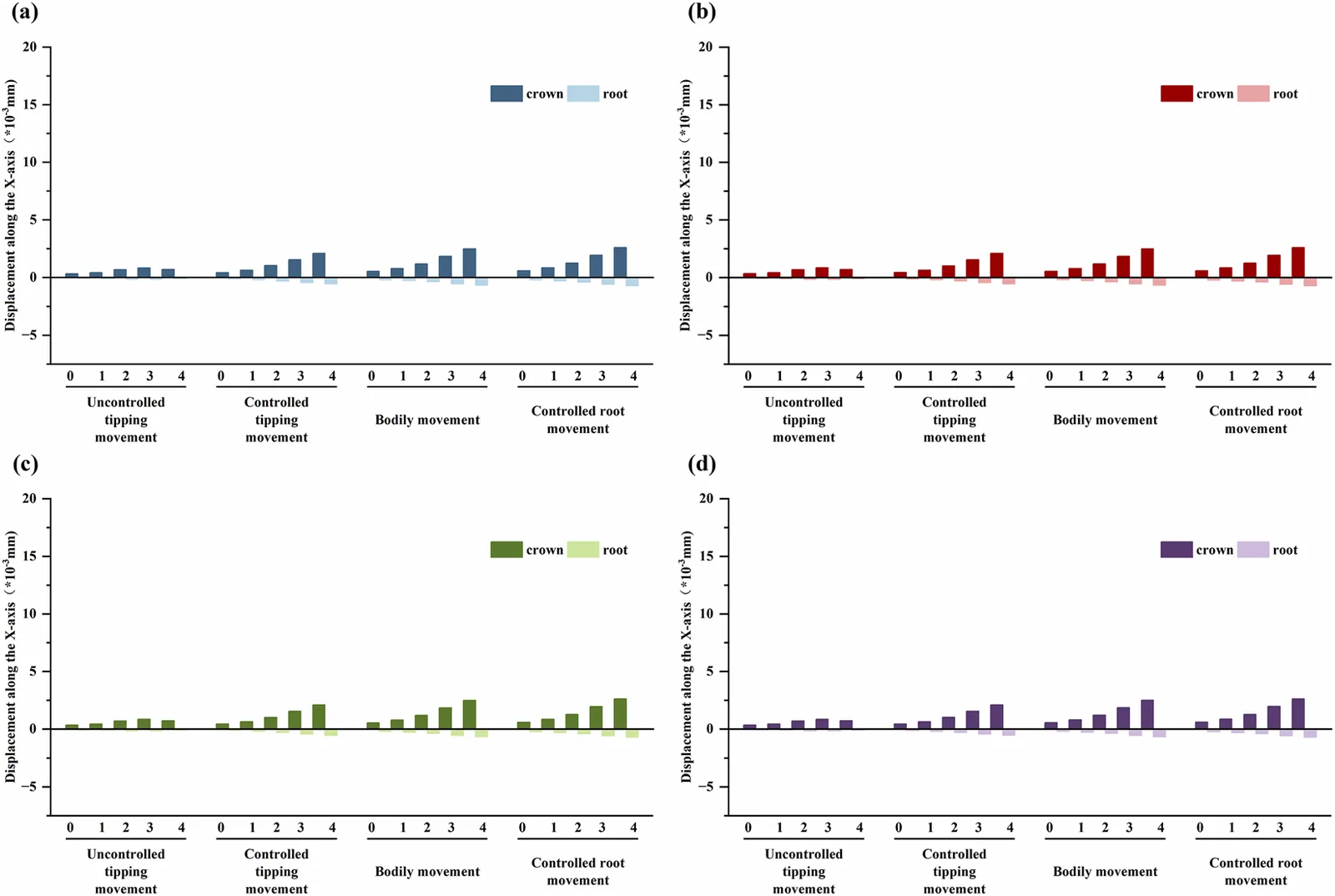
Displacement of the maxillary central incisor along the X-axis under five degrees of alveolar bone height loss (0, 1, 2, 3, 4 mm) and four types of tooth movement. **(a–d)** represent alveolar bone density levels I–IV.

**FIGURE 6:**
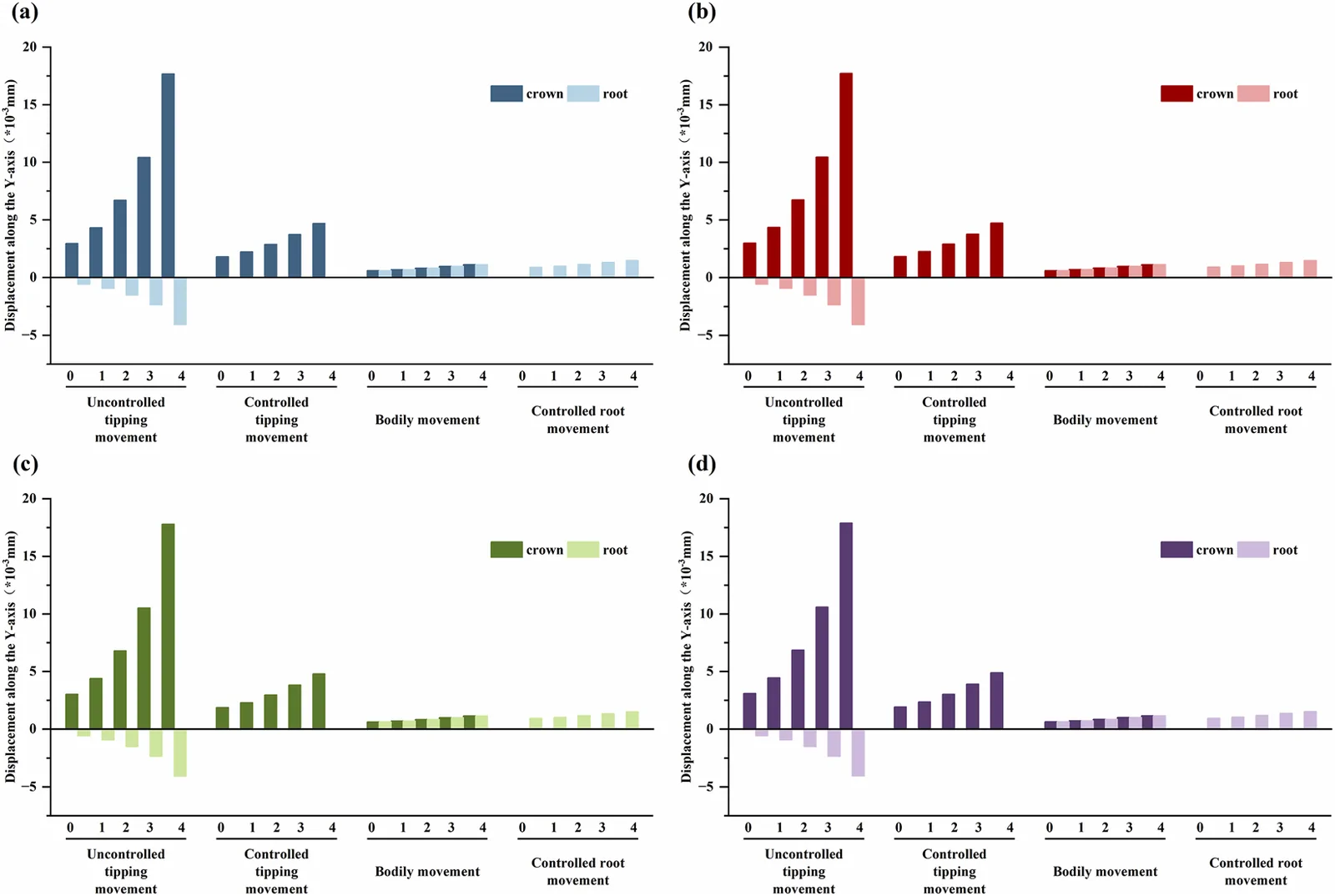
Displacement of the maxillary central incisor along the Y-axis under five degrees of alveolar bone height loss (0, 1, 2, 3, 4 mm) and four types of tooth movement. **(a–d)** represent alveolar bone density levels I–IV.

**FIGURE 7:**
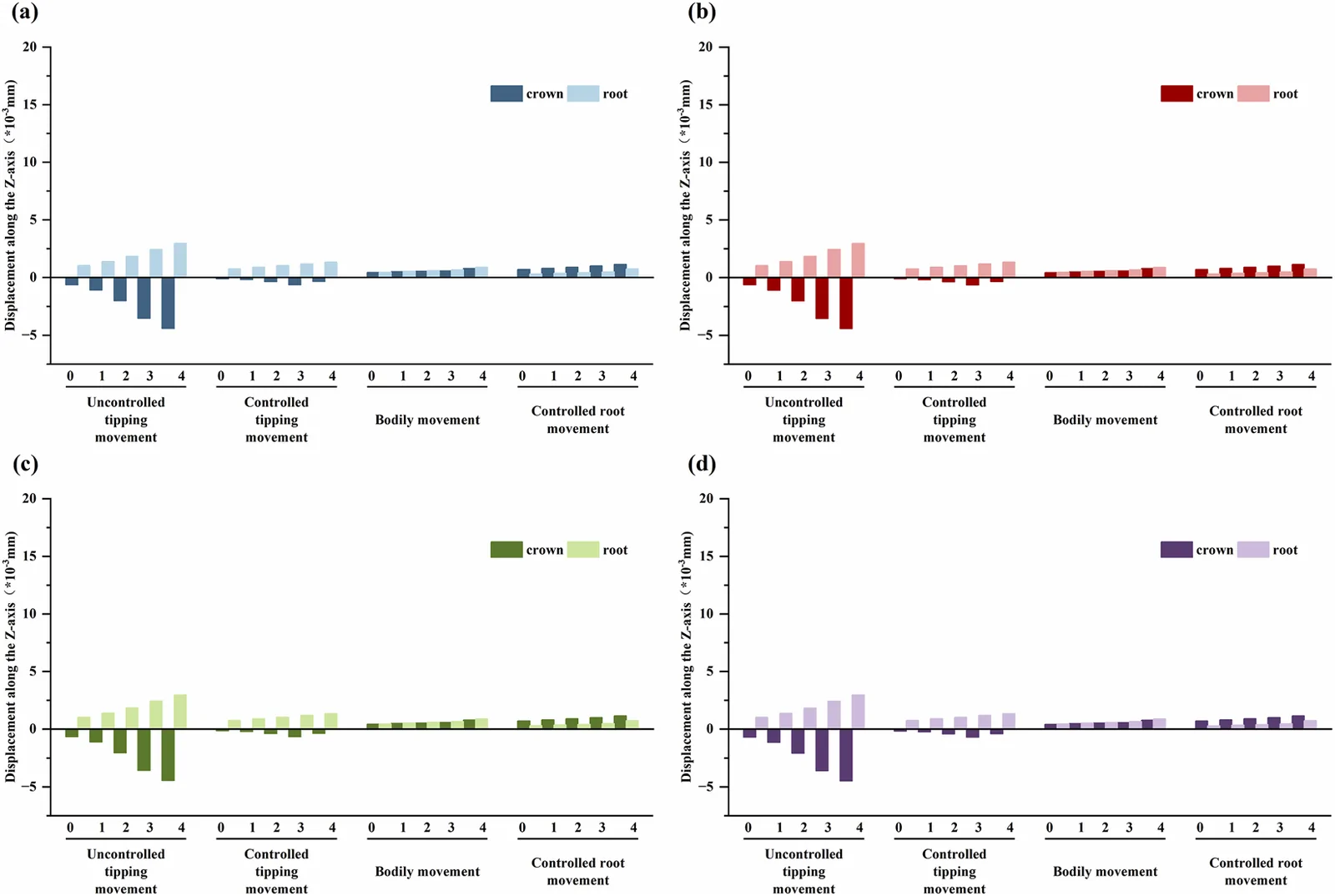
Displacement of the maxillary central incisor along the Z-axis under five degrees of alveolar bone height loss (0, 1, 2, 3, 4 mm) and four types of tooth movement. **(a–d)** represent alveolar bone density levels I–IV.

In the horizontal direction, with increasing alveolar bone height loss, both mesial crown tipping and distal root tipping showed increased magnitudes. Across all movement types, the rank order of displacement magnitudes for both crown and root was as follows: uncontrolled tipping < controlled tipping < bodily movement < controlled root movement. As alveolar bone density decreased, crown displacement showed a slight increasing trend, whereas root displacement remained relatively stable with only minor fluctuations (Figure 5; Supplementary Table S4). Sagittally, for each tooth movement type and across all alveolar bone density levels, the crown and root displacements of the maxillary central incisor increased with increasing alveolar bone height loss. Across all working conditions, the rank order of displacement magnitudes remained consistent: uncontrolled tipping produced the largest displacements, followed by controlled tipping, controlled root movement, and bodily movement. As alveolar bone density decreased from type I to IV, crown displacement in uncontrolled tipping showed an increasing trend, whereas the absolute values of root displacement showed a decreasing trend. In the other three movement types, tooth displacement increased with decreasing alveolar bone density (Figure 6; Supplementary Table S5). Along the Z-axis, with increasing alveolar bone height loss, the magnitudes of both crown and root displacement increased under most working conditions. In uncontrolled and controlled tipping movements, the crown exhibited an extrusive tendency, with extrusion being greater in uncontrolled tipping. Crown intrusion was greater in controlled root movement than in bodily movement. The root exhibited an intrusive tendency, with displacement magnitudes ranked as: uncontrolled tipping > controlled tipping > bodily movement > controlled root movement. In uncontrolled tipping, crown extrusion was smaller than root intrusion when bone loss was below 2 mm, but exceeded root intrusion when bone loss was at least 2 mm. In controlled tipping, root intrusion consistently exceeded crown extrusion, whereas in bodily movement, crown and root intrusion were comparable. Finally, in controlled root movement, crown intrusion consistently exceeded root intrusion. As bone density decreased, in uncontrolled and controlled tipping movements, the extrusive tendency of the crown increased while the intrusive tendency of the root decreased; in bodily and controlled root movements, the intrusive tendencies of both crown and root decreased (Figure 7; Supplementary Table S6).

### 3.2 Biomechanical performance of the PDL in the maxillary central incisor

**FIGURE 8:**
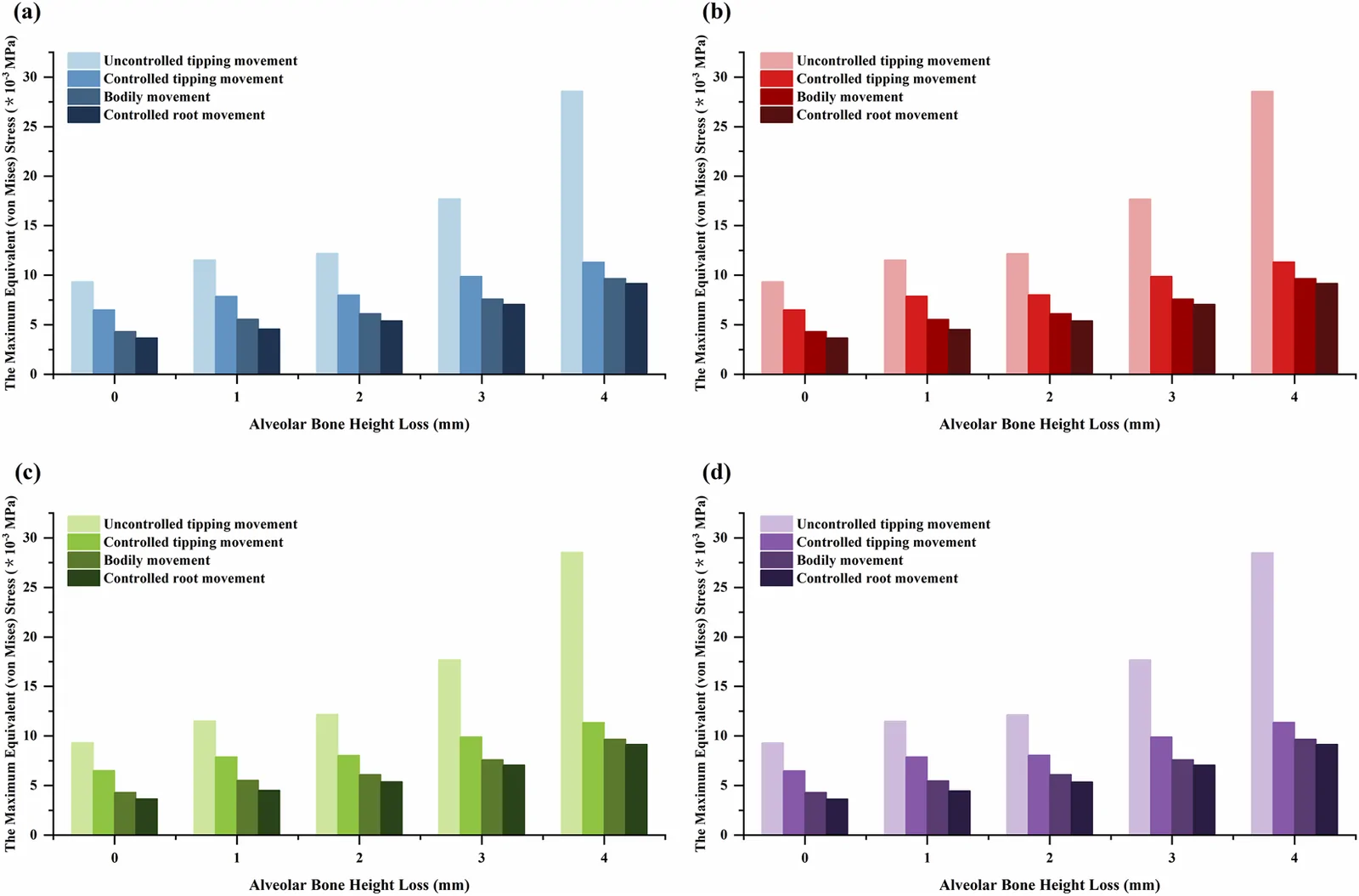
The maximum von Mises Stress (×10^−3^ MPa) in the PDL of the maxillary central incisor under five degrees of alveolar bone height loss (0, 1, 2, 3, 4 mm) and four types of tooth movement. **(a–d)** represent alveolar bone density levels I–IV.

**FIGURE 9:**
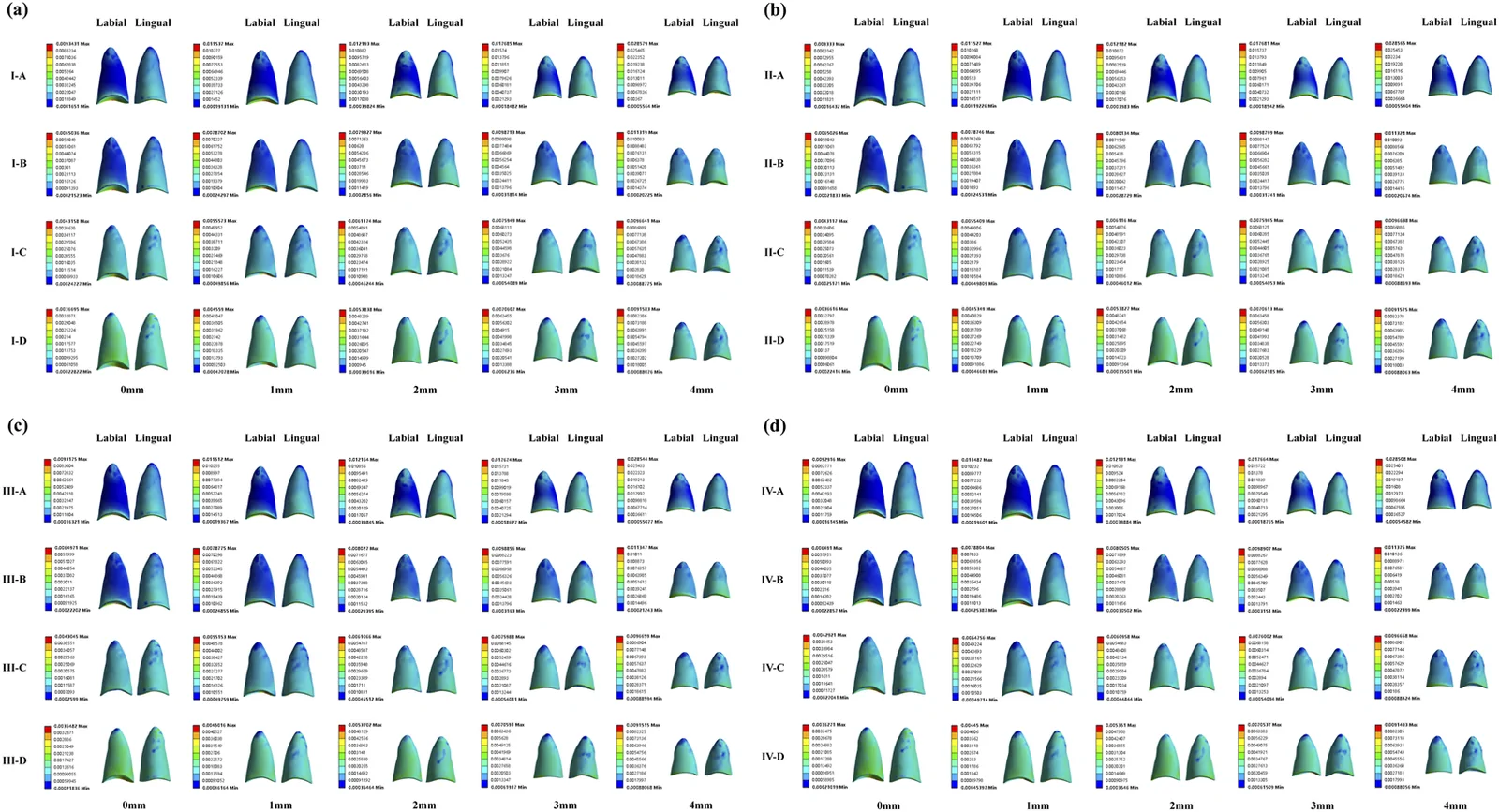
Contour plots of von Mises stress on the labial and lingual surfaces of the maxillary central incisor PDL under 80 loading conditions. Labels of 0, 1, 2, 3, and 4 mm denote the degree of alveolar bone height loss. **(a–d)** correspond to alveolar bone density levels I to IV, respectively. Within each panel, subpanels A–D represent uncontrolled tipping, controlled tipping, bodily movement, and controlled root movement, respectively.

The maximum von Mises stress values, which served as a comprehensive indicator in the PDL, are presented in Figure 8 and Supplementary Table S7; these values increased progressively with greater alveolar bone height loss. Across the four movement types, the maximum von Mises stress values in the PDL ranked in descending order as follows: uncontrolled tipping > controlled tipping > bodily movement > controlled root movement. In controlled tipping, the maximum von Mises stress increased with decreasing alveolar bone density when alveolar bone height loss reached at least 1 mm. In bodily movement, a similar increase was observed when alveolar bone height loss reached at least 3 mm. However, this trend was not evident in the other movement conditions. In the contour plots of the maximum von Mises stress (Figure 9), red and yellow indicated high-stress regions, whereas blue and green indicated low-stress regions. Across all contour plots, blue and green were predominant, suggesting that the overall stress levels within the PDL were relatively low. The maximum stress values were consistently observed in the lingual cervical region, and no extensive regions of stress concentration were identified.

### 3.3 Multiple linear regression analysis and equation

As shown in Supplementary Table S8, the multiple linear regression model yielded an R^2^ of 0.80, indicating that the independent variables (alveolar bone height loss, tooth movement types, and alveolar bone density) collectively explained 80% of the variance in the dependent variable (PDL stress), and demonstrating a good and reliable model fit. The regression model was statistically significant (F = 41.204, *p* < 0.05), demonstrating that at least one of the independent variables had a significant effect on the dependent variable.

Among the three independent variables, alveolar bone height loss (B = 2.066, β = 0.535, *p* < 0.05) was a significant predictor of PDL stress during anterior retraction and showed a positive effect. Tooth movement type was also a significant predictor of PDL stress. Compared with uncontrolled tipping movement, controlled tipping movement, bodily movement, and controlled root movement were associated with significantly lower PDL stress (all *p* < 0.05), with reductions of 7.122 × 10^−3^ MPa, 9.206 × 10^−3^ MPa, and 9.896 × 10^−3^ MPa respectively. Alveolar bone density was not a significant predictor, as no statistically significant differences were found between the moderately high-density (B = −0.003, β = 0.000, *p* > 0.05), moderately low-density (B = −0.010, β = −0.001, *p* > 0.05), and low-density groups (B = −0.022, β = −0.002, *p* > 0.05) compared with the high-density reference group. The regression equation was presented as follows (PDL stress and the intercept are in units of ×10^−3^ MPa; alveolar bone height loss is in units of mm): *PDL stress = 11.722 + 2.066 × (alveolar bone height loss) − 7.122 × (Controlled tipping movement) − 9.206 × (Bodily movement) − 9.896× (Controlled root movement).* All tooth movement types were dimensionless dummy variables (1 for the corresponding movement type, 0 for the reference group of uncontrolled tipping movement). In the regression equation, the standardized coefficients (*β*) of alveolar bone height loss, controlled tipping movement, bodily movement, and controlled root movement were 0.535, −0.564, −0.729, and −0.784, respectively. Therefore, among the independent variables, controlled root movement exerted the most significant influence on PDL stress, followed by bodily movement, controlled tipping movement, and alveolar bone height loss in descending order of importance. The Durbin–Watson statistic (1.407) suggested no autocorrelation in the residuals, and all variance inflation factor values were less than 5, indicating no multicollinearity among independent variables.

## 4 Discussion

The present study demonstrated that alveolar bone height loss, tooth movement types, and alveolar bone density exerted combined effects on the 3D displacements of the maxillary central incisor during initial retraction. Greater alveolar bone height loss was associated with increased 3D displacement of both the crown and root, an effect that can be attributed to the apical shift of the center of resistance resulting from alveolar bone height loss (Tanne et al., 1991). The magnitude of this displacement varied significantly across tooth movement types. Specifically, in uncontrolled tipping, crown sagittal displacement at 4 mm of alveolar bone height loss was approximately six times greater than at 0 mm, whereas the increases in controlled tipping, bodily movement, and controlled root movement were more moderate. Notably, in uncontrolled tipping, when alveolar bone height loss was below 2 mm, crown extrusion was less than root intrusion. However, when alveolar bone height loss reached ≥2 mm, crown extrusion predominated due to exponential increases in crown lingual tipping. Across all levels of alveolar bone height loss, uncontrolled and controlled tipping were characterized by substantial sagittal retraction, pronounced root intrusion, corresponding crown extrusion, and minimal distolingual rotation. In contrast, bodily movement and controlled root movement resulted in less sagittal retraction and root intrusion but greater distolingual rotation. Root intrusion and rotation consistently occurred together regardless of movement types, and in controlled root movement, crown intrusion consistently exceeded root intrusion. This difference can be attributed to an additional intrusive effect on the crown induced by traction during lingual root movement. This is consistent with findings by Rime et al., showing that increased torque led to incisal edge intrusion manifesting as labial inclination of the anterior teeth (Zalaquett et al., 2024). Additionally, decreased alveolar bone density also resulted in greater retraction, more pronounced distolingual rotation, and more prominent root intrusion. Alveolar bone density also differentially modulated the moments required for different movement types. Specifically, for controlled tipping, the shorter force arm and reduced alveolar bone density decreased alveolar constraint, thereby lowering the required moment. For bodily and controlled root movement, however, the longer force arm and the need for precise root control required greater torque to counteract the buffering effect of reduced bone stiffness. Collectively, these findings demonstrated that alveolar bone height loss, tooth movement type, and alveolar bone density exerted combined effects on both the magnitude and the characteristic pattern of the 3D displacement of the maxillary central incisor during initial retraction. Greater bone height loss and lower bone density amplify displacement, but this amplification is distinctly modulated by the specific tooth movement type.

Lee has reported a threshold value of 26 × 10^−3^ MPa for maximum stress in the PDL, although the specific stress type was not specified (Lee, 1965). In the present study, we measured maximum von Mises stress; therefore, Lee’s threshold was used only as a reference for trend-based comparison, and not as a direct indicator of mechanical safety. Under most working conditions, our calculated von Mises stresses fell below this reference value, with the exception of 4 mm alveolar bone loss combined with uncontrolled tipping, which exceeded the threshold across all bone density levels. Furthermore, the multiple linear regression results indicated that tooth movement type exerted the greatest negative influence on the maximum von Mises stress—that is, as the M/F ratio increased (from uncontrolled tipping to controlled tipping, bodily movement, and controlled root movement), the maximum stress decreased significantly.

Second, alveolar bone height loss exerted a significant positive effect on the maximum von Mises stress, indicating that greater alveolar bone height loss was associated with higher stress in the PDL. Increased alveolar bone height loss reduced the effective PDL area, thereby limiting the boundary of tooth movement. Under the same loading conditions, the increased load per unit area of the PDL resulted in stress concentration (Chen and Tsai, 2011). Finally, alveolar bone density did not display a significant effect on maximum von Mises stress during maxillary anterior tooth retraction. Consistently, Kuc et al. have reported that changes in alveolar bone density do not significantly affect PDL stress in the incisor region, suggesting that this region is less influenced by bone structure (Kuc et al., 2025).

In clinical practice, tooth movement typically involves a combination of movement types. For patients with Angle Class II, division 2 or Angle Class III malocclusion with excessively upright incisors, initial uncontrolled tipping with a round archwire may be used to restore normal labial inclination. For patients with excessively labially inclined anterior teeth but roots located centrally in the basal bone, controlled tipping is preferred during the early stage of retraction to prioritize crown movement and avoid premature contact between the root and the labial cortical bone. This should be followed by a transition to bodily movement at a later stage. Bodily movement is preferred in patients with skeletal protrusion and normal labial inclination of the anterior teeth, because it prevents deep overbite from excessive retraction and reduces stress concentration, thereby lowering the risk of alveolar crest and root resorption. When CBCT shows that the root is positioned too close to the labial cortical bone during retraction, controlled root movement is required. For patients with alveolar bone height loss, bodily movement should be delivered with a higher moment and a lower retraction force. Further adjustments should be made based on individual conditions, for instance, through adjunctive devices such as segmental arches and micro-implant anchorage (Sung et al., 2010). Additionally, moments can be preprogrammed into brackets or incorporated into the archwire to counteract adverse intrusion and rotation associated with increased alveolar bone height loss (Geramy, 2000). The follow-up interval should also be shortened to prevent excessive tooth movement (Artun and Urbye, 1988). Therefore, careful selection of the central incisor movement type and assessment of alveolar bone height loss are critical during the initial phase of anterior retraction. These findings provide quantitative biomechanical evidence for personalized movement pattern selection, enabling clinicians to match movement types to specific malocclusion features and to assess bone height loss levels to formulate individualized treatment plans. These quantitative relationships can also be incorporated into computer-assisted clinical decision-support systems. By integrating the regression model-derived weighting coefficients, a prediction module could automatically predict PDL stress distribution for a given set of patient-specific inputs (bone height, desired movement type). This would assist clinicians in pre-treatment risk assessment and treatment planning, and ultimately lay the foundation for accurate prediction of optimal orthodontic force magnitudes in the future, thereby minimizing PDL stress and its associated periodontal risks (Jain et al., 2021).

Previous studies have observed that dynamically remodeling alveolar bone density decreases by an average of 24% after 7 months of fixed orthodontic treatment, with the greatest reduction (29%) occurring around the maxillary central incisors (Hsu et al., 2011). Although the present study found no significant effect of central incisor bone density on initial PDL stress distribution during anterior retraction, pre-treatment CBCT assessment retains important prognostic value. Patients with lower pre-treatment bone density may exhibit impaired remodeling capacity and an increased risk of progressive bone loss and iatrogenic root resorption (Kuc et al., 2025). Therefore, as recommended by the American Academy of Periodontology consensus, pre-treatment CBCT assessment of periapical bone status is essential for identifying high-risk patients and for guiding individualized force adjustments to reduce the risk of periodontal complications (Mandelaris et al., 2017).

Nevertheless, this study has several limitations that should be acknowledged. First, clinical tooth movement is a dynamic biological process involving continuous remodeling of the tooth, PDL, and alveolar bone over time. Although static finite element analysis of initial displacement is a widely accepted approach in oral biomechanics, it cannot fully capture the time-dependent *in vivo* tissue responses (Kawamura et al., 2022). Second, the present finite element model adopts necessary simplifications for the orthodontic appliance system, and does not account for bracket–archwire contact conditions, ligation mechanics, slot–wire play, or torque expression efficiency, which may cause minor deviations between simulation results and actual *in vivo* biomechanical performance. Third, a well-validated single representative model was used to minimize thetableinfluence of anatomical confounding variables; however, this approach may limit the generalizability of our findings to populations with extreme anatomical variations. Finally, the multiple linear regression model primarily quantifies the linear contributions of individual variables and may not fully capture the complex non-linear interactions among these biomechanical factors. Therefore, our findings require further validation through well-designed animal experiments or prospective clinical trials.

To address these limitations, future research should prioritize three directions: (1) developing ML models based on large-scale parametric finite element datasets to capture complex non-linear biomechanical relationships; (2) integrating dynamic tissue remodeling algorithms into finite element simulations to improve prediction of long-term tooth movement; and (3) validating these computational findings in large clinical cohorts with diverse anatomical characteristics. These advances will ultimately pave the way for the development of more precise and personalized orthodontic treatment planning tools.

## 5 Conclusion

This study systematically investigated the combined effects of alveolar bone height loss, tooth movement types, and alveolar bone density on the initial retraction biomechanics of the maxillary central incisor. Using 80 working conditions and multiple linear regression, we analyzed 3D tooth displacement and quantified the weightings of these factors on PDL stress. These findings provide novel quantitative biomechanical evidence regarding the combined effects of these variables and can guide individualized force control strategies and the development of AI-assisted orthodontic decision-making systems.

## Data Availability

The original contributions presented in the study are included in the article/Supplementary Material, further inquiries can be directed to the corresponding author.
