# Supplementary material for: Combined effects and weight analysis of factors affecting initial maxillary central incisor retraction: a three-dimensional finite element study
Source: Front Bioeng Biotechnol. 2026 Sep 7;14:1895763. doi: 10.3389/fbioe.2026.1895763 (PMC13590767; doi:10.3389/fbioe.2026.1895763)
Supplement: Supplementary file 1 [file Table1.docx]

Table S1. Moment applied to the maxillary central incisors (N·mm)

| **Alveolar**  **bone density** | **Tooth movement type** | **Alveolar bone height loss** | | | | |
| --- | --- | --- | --- | --- | --- | --- |
|  |  | **0mm** | **1mm** | **2mm** | **3mm** | **4mm** |
| **I** | **Uncontrolled tipping movement** | 0 | 0 | 0 | 0 | 0 |
|  | **Controlled tipping movement** | 0.682 | 0.946 | 1.266 | 1.592 | 2.036 |
|  | **Bodily movement** | 1.390 | 1.646 | 1.939 | 2.249 | 2.597 |
|  | **Controlled root movement** | 1.744 | 1.960 | 2.204 | 2.481 | 2.773 |
| **II** | **Uncontrolled tipping movement** | 0 | 0 | 0 | 0 | 0 |
|  | **Controlled tipping movement** | 0.680 | 0.944 | 1.259 | 1.590 | 2.035 |
|  | **Bodily movement** | 1.396 | 1.651 | 1.943 | 2.252 | 2.600 |
|  | **Controlled root movement** | 1.753 | 1.968 | 2.215 | 2.485 | 2.776 |
| **III** | **Uncontrolled tipping movement** | 0 | 0 | 0 | 0 | 0 |
|  | **Controlled tipping movement** | 0.678 | 0.942 | 1.257 | 1.587 | 2.032 |
|  | **Bodily movement** | 1.405 | 1.659 | 1.949 | 2.257 | 2.603 |
|  | **Controlled root movement** | 1.767 | 1.979 | 2.224 | 2.492 | 2.782 |
| **IV** | **Uncontrolled tipping movement** | 0 | 0 | 0 | 0 | 0 |
|  | **Controlled tipping movement** | 0.674 | 0.939 | 1.253 | 1.584 | 2.028 |
|  | **Bodily movement** | 1.419 | 1.671 | 1.959 | 2.266 | 2.609 |
|  | **Controlled root movement** | 1.789 | 1.996 | 2.238 | 2.504 | 2.789 |
