# Supplementary material for: Combined effects and weight analysis of factors affecting initial maxillary central incisor retraction: a three-dimensional finite element study
Source: Front Bioeng Biotechnol. 2026 Sep 7;14:1895763. doi: 10.3389/fbioe.2026.1895763 (PMC13590767; doi:10.3389/fbioe.2026.1895763)
Supplement: Supplementary file 2 [file Table2.docx]

Table S2. Material properties used in finite element analyses

| **Material** | | **Young’s**  **modulus (MPa)** | **Poisson’s**  **ratio** |
| --- | --- | --- | --- |
| **Teeth** | | 19600 | 0.3 |
| **Periodontal ligament (PDL)** | | 0.69 | 0.45 |
| **Bracket, Archwire, hook** | | 200000 | 0.3 |
| **Cancellous bone** | | 1370 | 0.3 |
| **Cortical bone** | **I (High)** | 27500 | 0.3 |
|  | **II (Moderately high)** | 22500 | 0.3 |
|  | **III (Moderately low)** | 17500 | 0.3 |
|  | **IV (Low)** | 12500 | 0.3 |
