# Supplementary material for: Combined effects and weight analysis of factors affecting initial maxillary central incisor retraction: a three-dimensional finite element study
Source: Front Bioeng Biotechnol. 2026 Sep 7;14:1895763. doi: 10.3389/fbioe.2026.1895763 (PMC13590767; doi:10.3389/fbioe.2026.1895763)
Supplement: Supplementary file 3 [file Table3.docx]

|  | **Model A** | **Model B** | **Model C** | **Model D** | **Model E** |
| --- | --- | --- | --- | --- | --- |
| **Nodes** | 231049 | 222628 | 212409 | 203212 | 194526 |
| **Elements** | 121924 | 117223 | 111811 | 106645 | 102037 |

Table S3. Nodes and elements
