# Supplementary material for: Combined effects and weight analysis of factors affecting initial maxillary central incisor retraction: a three-dimensional finite element study
Source: Front Bioeng Biotechnol. 2026 Sep 7;14:1895763. doi: 10.3389/fbioe.2026.1895763 (PMC13590767; doi:10.3389/fbioe.2026.1895763)
Supplement: Supplementary file 4 [file Table4.docx]

Table S4. Displacement of maxillary central incisor (crown and root) along the X-axis（×10^-3^ mm)

| **Alveolar**  **bone density** | **Tooth movement type** | **Alveolar bone height loss** | | | | | | | | | |
| --- | --- | --- | --- | --- | --- | --- | --- | --- | --- | --- | --- |
|  |  | **0mm** | | **1mm** | | **2mm** | | **3mm** | | **4mm** | |
|  |  | **crown** | **root** | **crown** | **root** | **crown** | **root** | **crown** | **root** | **crown** | **root** |
| **I** | **Uncontrolled tipping movement** | 0.339 | -0.064 | 0.434 | -0.085 | 0.691 | -0.148 | 0.843 | -0.152 | 0.712 | -0.086 |
|  | **Controlled tipping movement** | 0.435 | -0.121 | 0.633 | -0.185 | 1.038 | -0.299 | 1.546 | -0.427 | 2.100 | -0.537 |
|  | **Bodily movement** | 0.536 | -0.181 | 0.780 | -0.259 | 1.185 | -0.357 | 1.836 | -0.540 | 2.483 | -0.661 |
|  | **Controlled root movement** | 0.586 | -0.211 | 0.846 | -0.292 | 1.260 | -0.392 | 1.938 | -0.580 | 2.603 | -0.700 |
| **II** | **Uncontrolled tipping movement** | 0.341 | -0.064 | 0.437 | -0.085 | 0.694 | -0.148 | 0.846 | -0.152 | 0.713 | -0.086 |
|  | **Controlled tipping movement** | 0.438 | -0.121 | 0.635 | -0.185 | 1.013 | -0.282 | 1.548 | -0.426 | 2.102 | -0.537 |
|  | **Bodily movement** | 0.539 | -0.181 | 0.784 | -0.259 | 1.190 | -0.358 | 1.840 | -0.540 | 2.487 | -0.662 |
|  | **Controlled root movement** | 0.590 | -0.212 | 0.850 | -0.293 | 1.260 | -0.388 | 1.943 | -0.581 | 2.608 | -0.701 |
| **III** | **Uncontrolled tipping movement** | 0.345 | -0.064 | 0.441 | -0.085 | 0.699 | -0.149 | 0.850 | -0.152 | 0.716 | -0.085 |
|  | **Controlled tipping movement** | 0.441 | -0.121 | 0.639 | -0.185 | 1.018 | -0.282 | 1.551 | -0.426 | 2.104 | -0.536 |
|  | **Bodily movement** | 0.544 | -0.183 | 0.789 | -0.261 | 1.197 | -0.359 | 1.847 | -0.541 | 2.493 | -0.663 |
|  | **Controlled root movement** | 0.595 | -0.213 | 0.857 | -0.294 | 1.268 | -0.390 | 1.951 | -0.582 | 2.616 | -0.702 |
| **IV** | **Uncontrolled tipping movement** | 0.351 | -0.065 | 0.446 | -0.085 | 0.706 | -0.149 | 0.856 | -0.152 | 0.720 | -0.085 |
|  | **Controlled tipping movement** | 0.446 | -0.121 | 0.644 | -0.185 | 1.024 | -0.282 | 1.557 | -0.425 | 2.107 | -0.535 |
|  | **Bodily movement** | 0.552 | -0.184 | 0.798 | -0.262 | 1.207 | -0.361 | 1.859 | -0.543 | 2.504 | -0.664 |
|  | **Controlled root movement** | 0.604 | -0.216 | 0.867 | -0.297 | 1.280 | -0.392 | 1.964 | -0.584 | 2.627 | -0.704 |
