# Supplementary material for: Combined effects and weight analysis of factors affecting initial maxillary central incisor retraction: a three-dimensional finite element study
Source: Front Bioeng Biotechnol. 2026 Sep 7;14:1895763. doi: 10.3389/fbioe.2026.1895763 (PMC13590767; doi:10.3389/fbioe.2026.1895763)
Supplement: Supplementary file 5 [file Table5.docx]

Table S5. Displacement of maxillary central incisor (crown and root) along the Y-Axis（×10^-3^ mm)

| **Alveolar**  **bone density** | **Tooth movement type** | **Alveolar bone height loss** | | | | | | | | | |
| --- | --- | --- | --- | --- | --- | --- | --- | --- | --- | --- | --- |
|  |  | **0mm** | | **1mm** | | **2mm** | | **3mm** | | **4mm** | |
|  |  | **crown** | **root** | **crown** | **root** | **crown** | **root** | **crown** | **root** | **crown** | **root** |
| **I** | **Uncontrolled tipping movement** | 2.965 | -0.582 | 4.320 | -0.936 | 6.703 | -1.525 | 10.423 | -2.363 | 17.681 | -4.084 |
|  | **Controlled tipping movement** | 1.806 | 2.6*10^-4^ | 2.236 | 6.6*10^-4^ | 2.872 | 5.2*10^-4^ | 3.735 | 1.2*10^-3^ | 4.701 | 7.6*10^-5^ |
|  | **Bodily movement** | 0.604 | 0.604 | 0.694 | 0.694 | 0.822 | 0.822 | 0.977 | 0.977 | 1.126 | 1.126 |
|  | **Controlled root movement** | 1.3*10^-3^ | 0.907 | 1.6*10^-3^ | 1.005 | 2.6*10^-3^ | 1.147 | 7.5*10^-4^ | 1.321 | 2.4*10^-3^ | 1.479 |
| **II** | **Uncontrolled tipping movement** | 2.991 | -0.581 | 4.349 | -0.935 | 6.736 | -1.524 | 10.460 | -2.361 | 17.723 | -4.081 |
|  | **Controlled tipping movement** | 1.832 | 2.1*10^-4^ | 2.264 | 3.1*10^-4^ | 2.912 | 7.2*10^-4^ | 3.770 | 9.4*10^-4^ | 4.735 | 1.7*10^-3^ |
|  | **Bodily movement** | 0.612 | 0.612 | 0.701 | 0.701 | 0.830 | 0.830 | 0.984 | 0.984 | 1.134 | 1.134 |
|  | **Controlled root movement** | 2.3*10^-3^ | 0.917 | 9.7*10^-4^ | 1.015 | 1.7*10^-3^ | 1.159 | 4.2*10^-3^ | 1.330 | 5.8*10^-3^ | 1.488 |
| **III** | **Uncontrolled tipping movement** | 3.028 | -0.579 | 4.391 | -0.934 | 6.783 | -1.522 | 10.513 | -2.358 | 17.785 | -4.076 |
|  | **Controlled tipping movement** | 1.867 | 7.7*10^-4^ | 2.301 | 5.9*10^-4^ | 2.954 | 1.2*10^-3^ | 3.821 | 2.1*10^-4^ | 4.795 | 6.6*10^-4^ |
|  | **Bodily movement** | 0.623 | 0.623 | 0.712 | 0.712 | 0.841 | 0.841 | 0.996 | 0.996 | 1.148 | 1.148 |
|  | **Controlled root movement** | 1.6*10^-3^ | 0.932 | 8.9*10^-4^ | 1.029 | 1.5*10^-3^ | 1.174 | 3.9*10^-3^ | 1.345 | 4.4*10^-4^ | 1.505 |
| **IV** | **Uncontrolled tipping movement** | 3.087 | -0.577 | 4.457 | -0.932 | 6.859 | -1.519 | 10.599 | -2.353 | 17.883 | -4.069 |
|  | **Controlled tipping movement** | 1.924 | 6.1*10^-4^ | 2.361 | 9.4*10^-4^ | 3.024 | 6.8*10^-4^ | 3.895 | 1.4*10^-3^ | 4.886 | 3.2*10^-4^ |
|  | **Bodily movement** | 0.639 | 0.639 | 0.728 | 0.728 | 0.858 | 0.858 | 1.014 | 1.014 | 1.165 | 1.165 |
|  | **Controlled root movement** | 5.8*10^-4^ | 0.956 | 1.7*10^-3^ | 1.051 | 2.3*10^-3^ | 1.196 | 3.6*10^-4^ | 1.369 | 8.1*10^-3^ | 1.527 |
