# Supplementary material for: Combined effects and weight analysis of factors affecting initial maxillary central incisor retraction: a three-dimensional finite element study
Source: Front Bioeng Biotechnol. 2026 Sep 7;14:1895763. doi: 10.3389/fbioe.2026.1895763 (PMC13590767; doi:10.3389/fbioe.2026.1895763)
Supplement: Supplementary file 6 [file Table6.docx]

Table S6. Displacement of maxillary central incisor (crown and root) along the Z-axis（×10^-3^ mm)

| **Alveolar**  **bone density** | **Tooth movement type** | **Alveolar bone height loss** | | | | | | | | | |
| --- | --- | --- | --- | --- | --- | --- | --- | --- | --- | --- | --- |
|  |  | **0mm** | | **1mm** | | **2mm** | | **3mm** | | **4mm** | |
|  |  | **crown** | **root** | **crown** | **root** | **crown** | **root** | **crown** | **root** | **crown** | **root** |
| **I** | **Uncontrolled tipping movement** | -0.617 | 1.042 | -1.073 | 1.385 | -2.007 | 1.843 | -3.526 | 2.446 | -4.412 | 2.970 |
|  | **Controlled tipping movement** | -0.099 | 0.760 | -0.166 | 0.902 | -0.342 | 1.042 | -0.617 | 1.192 | -0.330 | 1.343 |
|  | **Bodily movement** | 0.439 | 0.466 | 0.505 | 0.545 | 0.545 | 0.605 | 0.583 | 0.675 | 0.794 | 0.895 |
|  | **Controlled root movement** | 0.708 | 0.319 | 0.806 | 0.385 | 0.901 | 0.434 | 1.007 | 0.492 | 1.147 | 0.755 |
| **II** | **Uncontrolled tipping movement** | -0.632 | 1.039 | -1.088 | 1.383 | -2.025 | 1.840 | -3.545 | 2.443 | -4.428 | 2.967 |
|  | **Controlled tipping movement** | -0.113 | 0.757 | -0.180 | 0.901 | -0.364 | 1.037 | -0.635 | 1.191 | -0.343 | 1.342 |
|  | **Bodily movement** | 0.433 | 0.461 | 0.500 | 0.540 | 0.538 | 0.601 | 0.578 | 0.670 | 0.790 | 0.891 |
|  | **Controlled root movement** | 0.706 | 0.313 | 0.804 | 0.379 | 0.899 | 0.427 | 1.004 | 0.487 | 1.145 | 0.750 |
| **III** | **Uncontrolled tipping movement** | -0.654 | 1.035 | -1.110 | 1.379 | -2.050 | 1.836 | -3.573 | 2.439 | -4.452 | 2.963 |
|  | **Controlled tipping movement** | -0.133 | 0.754 | -0.200 | 0.899 | -0.386 | 1.034 | -0.660 | 1.190 | -0.364 | 1.341 |
|  | **Bodily movement** | 0.425 | 0.453 | 0.492 | 0.533 | 0.532 | 0.593 | 0.570 | 0.663 | 0.783 | 0.886 |
|  | **Controlled root movement** | 0.703 | 0.303 | 0.802 | 0.370 | 0.897 | 0.418 | 1.001 | 0.478 | 1.145 | 0.743 |
| **IV** | **Uncontrolled tipping movement** | -0.688 | 1.027 | -1.146 | 1.373 | -2.091 | 1.829 | -3.618 | 2.431 | -4.490 | 2.956 |
|  | **Controlled tipping movement** | -0.165 | 0.749 | -0.232 | 0.895 | -0.422 | 1.031 | -0.698 | 1.186 | -0.397 | 1.339 |
|  | **Bodily movement** | 0.412 | 0.441 | 0.481 | 0.522 | 0.520 | 0.581 | 0.559 | 0.650 | 0.774 | 0.876 |
|  | **Controlled root movement** | 0.699 | 0.288 | 0.798 | 0.356 | 0.892 | 0.404 | 0.999 | 0.463 | 1.139 | 0.732 |
