# Supplementary material for: Combined effects and weight analysis of factors affecting initial maxillary central incisor retraction: a three-dimensional finite element study
Source: Front Bioeng Biotechnol. 2026 Sep 7;14:1895763. doi: 10.3389/fbioe.2026.1895763 (PMC13590767; doi:10.3389/fbioe.2026.1895763)
Supplement: Supplementary file 7 [file Table7.docx]

Table S7. The maximum von Mises Stress in the PDL of the maxillary central incisor (×10^-3^ MPa)

| **Alveolar**  **bone density** | **Tooth movement type** | **Alveolar bone height loss** | | | | |
| --- | --- | --- | --- | --- | --- | --- |
|  |  | **0mm** | **1mm** | **2mm** | **3mm** | **4mm** |
| **I** | **Uncontrolled tipping movement** | 9.343 | 11.537 | 12.193 | 17.685 | 28.579 |
|  | **Controlled tipping movement** | 6.504 | 7.870 | 7.993 | 9.871 | 11.319 |
|  | **Bodily movement** | 4.316 | 5.557 | 6.117 | 7.595 | 9.664 |
|  | **Controlled root movement** | 3.670 | 4.559 | 5.384 | 7.061 | 9.158 |
| **II** | **Uncontrolled tipping movement** | 9.333 | 11.527 | 12.182 | 17.681 | 28.565 |
|  | **Controlled tipping movement** | 6.503 | 7.875 | 8.013 | 9.877 | 11.328 |
|  | **Bodily movement** | 4.312 | 5.541 | 6.113 | 7.597 | 9.664 |
|  | **Controlled root movement** | 3.662 | 4.535 | 5.383 | 7.061 | 9.158 |
| **III** | **Uncontrolled tipping movement** | 9.318 | 11.512 | 12.164 | 17.674 | 28.544 |
|  | **Controlled tipping movement** | 6.497 | 7.878 | 8.027 | 9.886 | 11.347 |
|  | **Bodily movement** | 4.305 | 5.515 | 6.107 | 7.599 | 9.666 |
|  | **Controlled root movement** | 3.648 | 4.502 | 5.370 | 7.059 | 9.152 |
| **IV** | **Uncontrolled tipping movement** | 9.292 | 11.487 | 12.131 | 17.664 | 28.508 |
|  | **Controlled tipping movement** | 6.491 | 7.880 | 8.051 | 9.891 | 11.375 |
|  | **Bodily movement** | 4.292 | 5.476 | 6.096 | 7.600 | 9.666 |
|  | **Controlled root movement** | 3.627 | 4.450 | 5.351 | 7.054 | 9.149 |
