# Supplementary material for: Combined effects and weight analysis of factors affecting initial maxillary central incisor retraction: a three-dimensional finite element study
Source: Front Bioeng Biotechnol. 2026 Sep 7;14:1895763. doi: 10.3389/fbioe.2026.1895763 (PMC13590767; doi:10.3389/fbioe.2026.1895763)
Supplement: Supplementary file 8 [file Table8.docx]

Table S8. Multiple linear regression analysis of factors influencing PDL von Mises Stress(×10⁻³ MPa) during maxillary central incisor retraction

| **Variable** | | **B** | **SE** | **Beta**  **(β)** | **t** | **P** | **Collinearity diagnosis** | |
| --- | --- | --- | --- | --- | --- | --- | --- | --- |
|  |  |  |  |  |  |  | **Tolerance** | **VIF** |
| **(Constant)** | | 11.722 | 0.864 |  | 13.571 | 0.000^*^ |  |  |
| **Alveolar bone height loss** | | 2.066 | 0.204 | 0.535 | 10.149 | 0.000^*^ | 1.000 | 1.000 |
| **Alveolar**  **bone density** | **I (High)** | 0 |  |  |  |  |  |  |
|  | **II (Moderately high)** | -0.003 | 0.814 | 0.000 | -0.004 | 0.997 | 0.667 | 1.500 |
|  | **III (Moderately low)** | -0.010 | 0.814 | -0.001 | -0.013 | 0.990 | 0.667 | 1.500 |
|  | **IV (Low)** | -0.022 | 0.814 | -0.002 | -0.027 | 0.978 | 0.667 | 1.500 |
| **Tooth movement type** | **Uncontrolled tipping movement** | 0 |  |  |  |  |  |  |
|  | **Controlled tipping movement** | -7.122 | 0.814 | -0.564 | -8.746 | 0.000^*^ | 0.667 | 1.500 |
|  | **Bodily movement** | -9.206 | 0.814 | -0.729 | -11.305 | 0.000^*^ | 0.667 | 1.500 |
|  | **Controlled root movement** | -9.896 | 0.814 | -0.784 | -12.152 | 0.000^*^ | 0.667 | 1.500 |
| **R^2^** | | 0.800 | | | | | | |
| **adjusted R^2^** | | 0.781 | | | | | | |
| **F** | | 41.204 P＜0.05 | | | | | | |
| **D-W** | | 1.407 | | | | | | |

^*^ P＜0.05
